# RESC: A Rubric-Guided Error–Score Consistency Framework for Action Quality Assessment in Vocational Fitter Training

**DOI:** 10.3390/s26175575

**Published:** 2026-09-02

**Authors:** Wenfeng Mu, Shigang Wang, Jiajia Huang

**Affiliations:** 1 School of Automation, Guangxi University of Science and Technology, Liuzhou 545006, China; mfkeyman@163.com (W.M.); 15078288942@163.com (J.H.); 2 Guangxi Low-Altitude Unmanned Aircraft Key Technologies Engineering Research Center, Liuzhou 545616, China

**Keywords:** action quality assessment, vocational fitter training, rubric-guided learning, human pose estimation, error diagnosis, interpretable scoring

## Abstract

Action quality assessment in vocational fitter training is constrained by limited expert-annotated data and insufficiently interpretable predictions. To achieve accurate scoring and rubric-aligned, auditable explanations with limited data, this study proposes RESC, a rubric-guided error–score consistency framework. RESC uses human pose as its primary visual evidence and converts an instructor rubric into error semantics and scoring constraints. It therefore predicts an overall score while producing rubric-aligned error diagnoses and model-derived itemized deduction contributions. We constructed Fitter-AQA, a dataset of 134 filing videos from 25 participants, and evaluated the framework using strict leave-one-subject-out cross-validation. RESC achieved a mean absolute error of 4.450, a Pearson linear correlation coefficient of 0.902, and a Spearman rank correlation coefficient of 0.852, corresponding to the lowest pooled scoring errors and highest pooled correlations among the evaluated methods. Error diagnosis reached a Macro-F1 of 0.770 and a macro-average balanced accuracy of 0.824. These results indicate stable cross-subject scoring under limited-data conditions and transform model predictions into an auditable score decomposition aligned with rubric semantics. RESC thus provides an accurate and interpretable approach to automated assessment in vocational skills training.

## 1. Introduction

Action quality assessment (AQA) quantifies how well a human action is performed. It extends deep learning-based human action analysis from category recognition to skill evaluation [1]. Unlike action recognition, which mainly determines what action is being performed, AQA must distinguish subtle differences in technical compliance and execution quality within the same action. These differences must then be translated into meaningful quality judgements. With advances in three-dimensional convolutional networks, spatiotemporal graph convolutional networks, and Transformers, RGB videos and human pose sequences have become the main inputs for AQA [2,3,4]. Contrastive regression, relative ranking, and procedural parsing further improve the representation of quality levels and local execution differences [5,6,7,8]. These advances have extended AQA from competitive sports to rehabilitation and professional skill assessment. Most deep learning-based AQA methods learn a direct mapping from full-video spatiotemporal representations to an overall score. This strategy captures stage relations and overall execution quality from continuous motion. However, in vocational training, video-level score supervision may encourage models to exploit quality-related patterns that lack instructional meaning. Differences in body shape, movement habits, and operating speed can further impair generalization to unseen trainees, even when training-set fitting is satisfactory. A total score can indicate whether an action was performed well, but it cannot locate an error or quantify its severity and associated deduction. Therefore, vocational skill AQA requires both reliable cross-subject scoring and evaluation evidence that instructors and trainees can understand.

The collection and annotation of authentic vocational skill videos are constrained by workstation conditions, participant coordination, and the cost of expert assessment [9,10,11,12,13]. Unlike isolated actions that can be repeatedly recorded under standardized conditions, manual filing in fitter training involves continuous interaction between the body and the tool. The recording process is also affected by workstation occlusion, lighting variation, and individual differences in stance. Quality differences are often concentrated in local body cues. Consequently, each error category contains relatively few valid samples, and repeated recording does not readily produce a balanced data distribution. Limited sample size is therefore an inherent characteristic of industrial training data. Under these conditions, high-capacity models are more likely to learn incidental correlations with body shape, clothing, or the recording environment. Strict cross-subject evaluation can thus better reflect generalization to new trainees than random data splitting. Automated assessment in vocational training also serves a different instructional purpose from conventional video regression. If a model predicts only an overall score, it is difficult to determine whether that prediction is based on the correct motion evidence. Attention maps and key-segment visualizations may indicate where the model focuses, but they do not directly correspond to rubric items or deductions. Converting the assessment rubric into a structured prior can narrow the evaluation space that the model must discover independently. It can also establish an auditable relationship between identified errors and the final score.

To address these questions, we propose RESC, a rubric-guided error–score consistency framework. RESC begins with human keypoint sequences extracted by deep pose estimation and introduces the instructor rubric as a structured prior for quality modeling. This design focuses the model on posture evidence directly associated with operating standards and explains the overall score through rubric-aligned diagnoses and a model-derived deduction decomposition. Task knowledge is used to reduce learning difficulty under limited-data conditions while supporting model inspection and human review.

The main contributions of this study are as follows:

1. We propose RESC, a rubric-guided AQA framework for small-sample vocational training. It combines deep pose representations with instructor scoring rules to improve cross-subject scoring and provide traceable error evidence and model-derived deduction contributions for the final score.
2. We build a video acquisition system in an authentic training workshop and construct the Fitter-AQA dataset. The dataset contains 134 operation videos from 25 participants with different skill levels and provides instructor-consensus scores, error categories, and severity labels for small-sample AQA research in realistic industrial training environments.
3. We systematically evaluate scoring, ranking, and error diagnosis under strict leave-one-subject-out cross-validation. Comparisons with feature-learning, elastic-alignment, and rubric-guided methods, together with multiple ablation studies, demonstrate the value of structured rubric priors under limited-data conditions.

## 2. Related Work

### 2.1. Deep-Learning-Based Action Quality Assessment

Deep-learning-based AQA typically encodes a continuous video or skeleton sequence into a spatiotemporal representation and then estimates quality through regression, ranking, or score-distribution prediction. Early studies mainly used video-level convolutional features and recurrent networks [14,15,16,17]. SlowFast networks, spatiotemporal attention, graph convolution, and Transformers subsequently improved the modeling of long-range dependencies and inter-joint relationships [4,18,19,20,21,22]. Score supervision has also progressed from absolute score regression to pairwise ranking, group-aware contrastive regression, and uncertainty-aware distribution learning [5,6,23,24]. Deep spatiotemporal models can learn complex quality patterns from large video collections, but their performance depends on sufficiently representative training data. Recent skeleton-based AQA research likewise identifies annotation dependence and cross-domain generalization as key constraints on deployment [25]. When the training set is small and subject variation is pronounced, increased model capacity does not necessarily yield stable gains. Task-oriented representations and prior constraints remain important for improving sample efficiency.

### 2.2. Limited-Sample Learning and Cross-Subject Generalization

Unlike large action-recognition datasets, task-specific AQA datasets rarely contain abundant videos with expert labels. Models are therefore more vulnerable to subject appearance, scene background, and a small number of atypical samples. Early studies used segment-level training and pretrained spatiotemporal features to ease optimization with limited data [15]. In professional skill assessment, Yanik et al. used meta-learning for one-shot or few-shot adaptation across surgical tasks [26]. Related few-shot action-recognition studies improved the use of scarce examples through prototype-centered representations [27] and two-stage temporal alignment [28]. These findings show that transferring existing representations and explicitly modeling sample relationships can reduce data requirements. However, current work focuses mainly on novel-class recognition or cross-task skill-level transfer, with less attention to continuous quality scoring under strict cross-subject protocols. Limited-sample AQA for professional skills must suppress identity-related bias while retaining quality information aligned with evaluation rules. This study therefore examines whether structured rubric priors improve cross-subject scoring stability.

### 2.3. Pose-Aware and Fine-Grained Process Modeling

Human pose estimation converts RGB video into keypoint sequences with an explicit body topology, offering a compact representation that is less sensitive to background interference. Skeleton-based graph neural networks model spatial joint relations and their temporal evolution [4,22], while joint angles, relative distances, and motion trajectories describe local operating standards in vocational tasks [29,30,31,32,33,34]. Other studies improve fine-grained understanding by modeling action stages and human-centered regions. FineDiving organizes complete actions using procedural semantics [7,35], whereas FineParser, TSA-Net, and the Temporal Parsing Transformer identify score-relevant processes through spatiotemporal parsing, local attention, and stage relations [8,20,21]. These studies show that pose structure and process decomposition help distinguish quality differences among visually similar videos. Nevertheless, a key segment or attended region does not directly identify the violated operating criterion or quantify its effect on the final score. Pose evidence must be linked to domain evaluation rules before it can support rubric-aligned diagnosis.

### 2.4. Rubric-Driven Interpretable Scoring

Interpretability research in AQA is shifting from post hoc attention visualization toward intrinsic explanations directly associated with scoring semantics. Gradient attribution, concept activation, and prototype learning identify important regions or intelligible concepts [36,37,38,39]. Fine-grained parsing and causal modeling further reduce background interference and reveal spatiotemporal evidence that affects scores [7,8,20,21,40]. For professional evaluation, IRIS organizes action segments and subscore explanations using a scoring rubric [41]; RICA^2^ encodes action steps and scoring criteria as structured relations [42]; and NS-AQA and RCS generate fine-grained assessments from expert rules or natural-language rubrics [43,44]. These studies demonstrate the value of domain rules for trustworthy AQA. Most, however, address phases, technical elements, or subscores in competitive actions. Itemized error diagnosis and deduction consistency in small-sample vocational training remain underexplored. RESC uses rubric priors to improve cross-subject scoring and interpretability, while monotonic constraints align changes in error severity with changes in deduction magnitude [45,46,47].

## 3. Materials and Methods

### 3.1. Data Acquisition System

**Figure 1:**
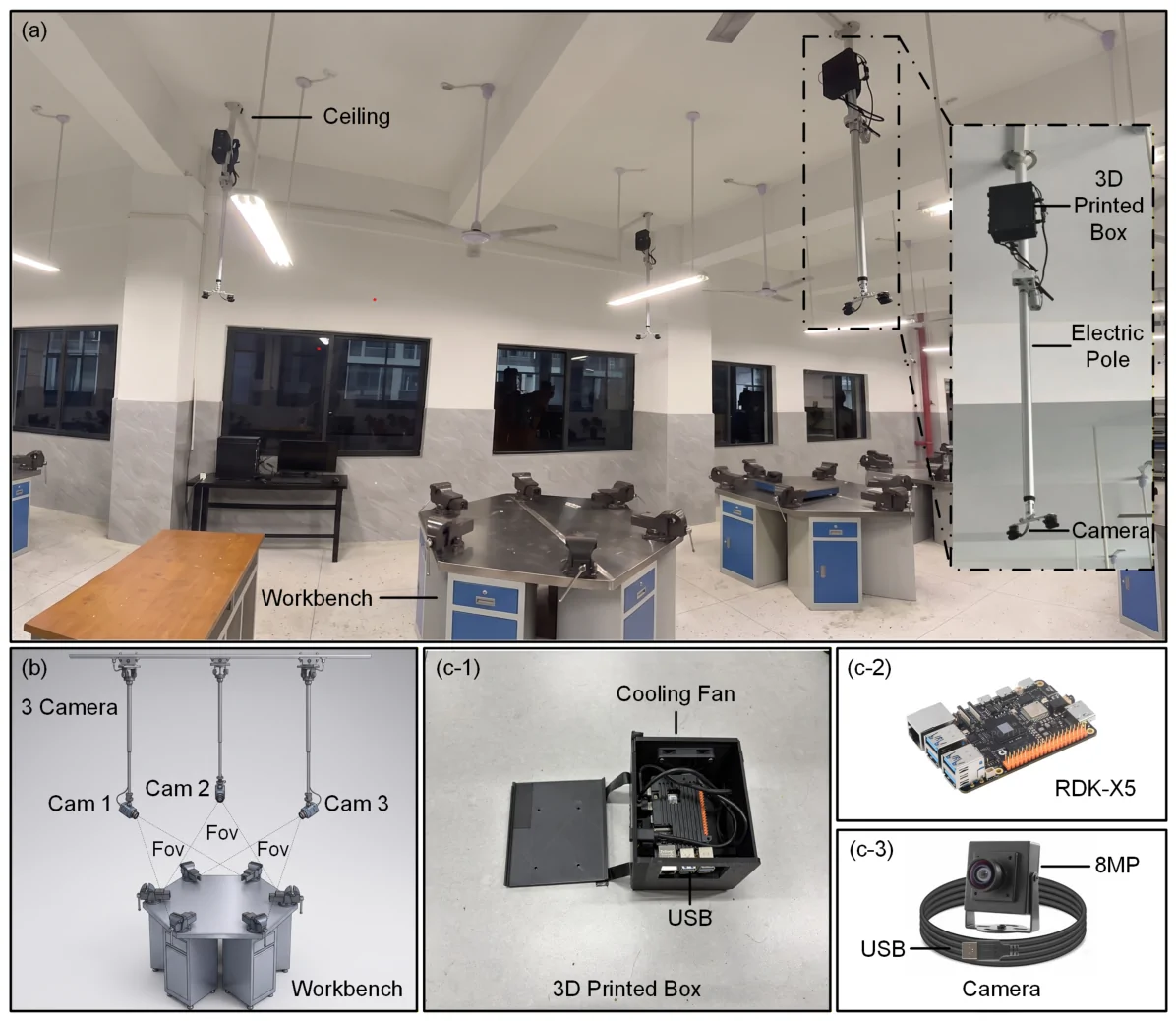
(**a**) Video environment and an enlarged view of the acquisition unit; (**b**) installation layout; and (**c**) hardware components, including (**c-1**) the 3D-printed enclosure integrating the RDK-X5 board, power supply, and cooling system, (**c-2**) the low-cost RDK-X5 edge-computing board (Shenzhen D-Robotics Co., Ltd., Shenzhen, China), and (**c-3**) the 8-megapixel USB industrial camera (Shenzhen Jierui Weitong Electronic Technology Co., Ltd., Shenzhen, China).

We installed a fixed overhead acquisition system in a vocational fitter training workshop to record authentic filing practice. The acquisition area covered both the workbench and the trainee’s standing space, allowing the system to capture the torso and the filing stroke. A ceiling-mounted camera reduced scale changes and viewpoint disturbances and minimized interference with normal practice. Figure 1 presents the system configuration. Three identical acquisition units were installed above one group of workbenches, with overlapping overhead fields of view covering six workstations. Each unit was suspended from the ceiling by a motorized boom, and the edge-computing board transmitted the video stream over Wi-Fi. The fixed layout maintained consistent acquisition conditions while retaining realistic disturbances, including occlusion by tools and benches, illumination variation, and partial pose invisibility. Video-level camera and workstation identifiers were not retained during the original collection, and participants were not assigned to balanced acquisition-unit strata. The acquisition unit associated with an individual video therefore cannot be reconstructed from the current metadata.

### 3.2. Dataset Construction and Quality Control

**Figure 2:**
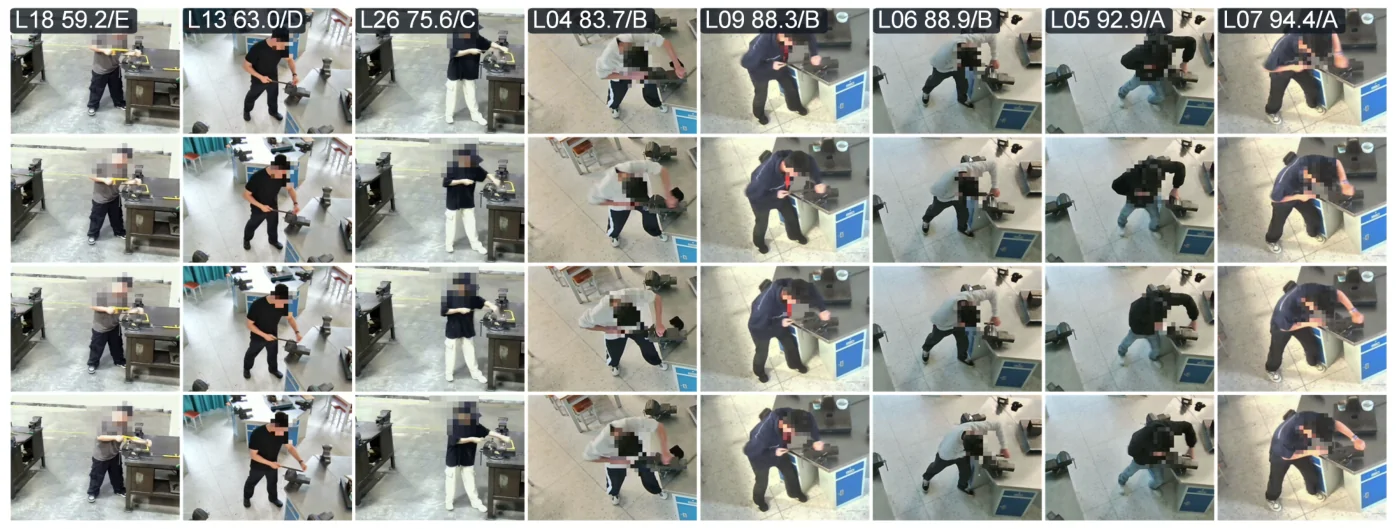
Examples from the Fitter-AQA dataset. Videos from eight participants are ordered from lower to higher expert scores. Each column shows four temporal positions from one video, and the participant identifier, expert score, and quality level are shown above the column.

The acquisition system was used to collect and curate filing-practice videos from beginners, award-winning skills competitors, and experienced instructors. Each video was reviewed for decodability and action completeness. The resulting Fitter-AQA dataset contains 134 videos from 25 participants, with a mean duration of 30.8 s. Individual participants contributed between 1 and 7 videos, with a median of 2 videos per participant. Four instructors experienced in vocational fitter training jointly reviewed each video using a common rubric. Each of the six errors was assigned a four-level ordinal label from 0 to 3, with severity determined according to the magnitude, persistence, and functional influence of the observed deviation. Error occurrence, severity, and the overall score were determined collectively by the four instructors. These consensus annotations supervise the model-derived itemized deduction contributions because the instructional rubric assigns the final score holistically. The operational definitions and corresponding pose evidence are summarized in Table 1. Scores ranged from 53.2 to 100, with a mean of 79.76 and a standard deviation of 13.35. For auxiliary quality-level supervision, scores were categorized as level A (≥90), level B (80–89.9), level C (70–79.9), level D (60–69.9), and level E (<60). We did not resample quality levels, thereby preserving the natural score distribution of authentic instruction. All subsequent experiments used this dataset. Representative samples are shown in Figure 2.

### 3.3. Task Definition and Overview of RESC

Action quality assessment in vocational training involves score prediction, error recognition, and deduction explanation. Instructors typically inspect key postures and the operation process, identify error types, and then assign a quality score. We therefore formulate automated AQA as a rubric-evidence-driven multi-output inference problem. Given the *i*th video $V_{i}$ and an expert rubric R, the model jointly outputs an overall score, probabilities for six errors, their severities, adaptive deduction gates, and model-derived itemized deduction contributions:

(1) $$\left({\hat{y}}_{i},p_{i},{\hat{s}}_{i},{\hat{r}}_{i},{\hat{d}}_{i}\right)=F_{\Theta }\left(V_{i},R\right).$$

**Figure 3:**
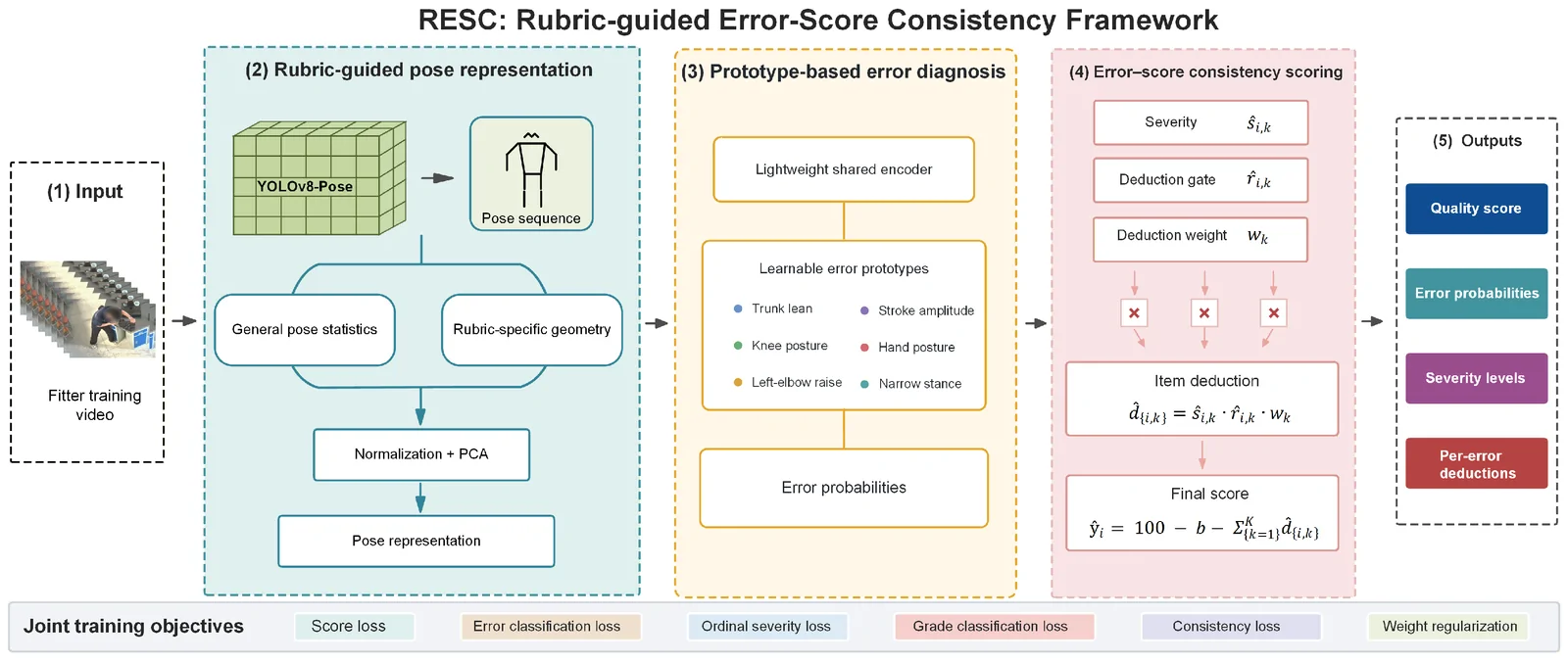
Overview of RESC. Skeleton features are first extracted and concatenated. Rubric-aligned error prototypes then diagnose the six error categories. Their model-derived severity-dependent deduction contributions are aggregated to produce the quality score, together with error categories, severity levels, and itemized deductions.

Here, ${\hat{y}}_{i}$ is the predicted overall score, and K=6 is the number of error categories. The *K*-dimensional vectors $p_{i}$, ${\hat{s}}_{i}$, ${\hat{r}}_{i}$, and ${\hat{d}}_{i}$ contain the error probabilities, expected severities, adaptive deduction gates, and model-derived itemized deduction contributions, respectively. Their components are indexed by k=1,…,K. The itemized contributions are estimated from the instructor-assigned overall score under joint error-category and severity supervision. An overview of the proposed RESC framework is illustrated in Figure 3.

### 3.4. Rubric-Guided Pose Geometry

The main quality differences in filing are reflected in body support, trunk stability, elbow–wrist relations, and the reciprocating filing trajectory. We therefore use pose geometry as the primary visual evidence. For each video $V_{i}$, T=64 temporal positions are sampled at equal normalized intervals over the complete valid timeline, providing coverage of the full operation. YOLOv8-Pose then extracts J=17 body keypoints and their confidence scores. The pose sequence is defined as

(2) $$P_{i}=\left\{\left(u_{i,t,j},q_{i,t,j}\right)|t=1,\ldots ,T;\;j=1,\ldots ,J\right\},\;J=17,$$

where *T* is the number of sampled time points and J=17 is the number of body keypoints. The vector $u_{i,t,j}$ is the two-dimensional coordinate of keypoint *j* at time *t* in video *i*, and $q_{i,t,j}$ is its confidence. We use $P_{i,t}={\left\{\left(u_{i,t,j},q_{i,t,j}\right)\right\}}_{j=1}^{J}$ to denote the frame-level pose at time *t*. Before sampling, the frame sequence is checked for validity. Frames that cannot be decoded, contain abrupt visual corruption, or clearly fall outside the effective operation are removed. Uniform sampling is then performed over the remaining valid interval. A keypoint with $q_{i,t,j}<0.20$ is treated as missing. Its normalized coordinates are encoded as zero, and temporal interpolation is not applied. If YOLOv8-Pose does not detect a valid person at a sampled position, the complete frame-level descriptor for that position is set to zero.

The pose representation comprises general pose statistics and category-specific rubric geometry. Filing is strongly periodic, so posture distributions and inter-sample changes are compressed into stable video-level statistics. This design reduces the risk of overfitting a high-capacity temporal model to limited data. Keypoint coordinates are first normalized to body scale. Relative distances, angles, displacements, and adjacent-sample changes among the shoulders, hips, elbows, wrists, knees, and ankles are then calculated. A statistical operator Γ aggregates the general pose descriptors:

(3) $$g_{i}^{\mathrm{base}}=\Gamma \left({\left\{\psi^{\mathrm{base}}\left(P_{i,t}\right)\right\}}_{t=1}^{T}\right),$$

(4) $$\Gamma =\left\{\mathrm{mean},\mathrm{std},\min,\max,Q_{25},Q_{50},Q_{75},\mathrm{mean}\left(|\Delta |\right),\mathrm{mean}\left(\Delta^{2}\right)\right\}.$$

Here, $\psi^{\mathrm{base}}$ denotes the general pose descriptor. The symbols $Q_{25}$, $Q_{50}$, and $Q_{75}$ denote its feature-wise 25th, 50th, and 75th percentiles. The operator Δ denotes the feature-wise first-order difference between descriptors at adjacent sampled times. Means and quantiles characterize typical posture, standard deviations and extrema describe stability and range, and the difference statistics capture local motion continuity. The implementation uses 28 frame-level general descriptors covering detection confidence, normalized body scale, joint angles, relative joint positions, and inter-joint distances. Their temporal statistics and four sequence-level summaries produce the 200-dimensional general-pose block. Appendix A, Table A2 provides the complete compact dimensional specification.

Rubric geometry explicitly encodes the body regions inspected by instructors. The keypoint sets for the six errors are

(5) $$J^{\mathrm{rubric}}=\left\{\begin{array}{cccc} J_{\mathrm{inc}} & =\{6,12,14,16\}, & J_{\mathrm{knee}} & =\{11,13,15\},\\ J_{\mathrm{elbow}} & =\{5,7,9\}, & J_{\mathrm{amp}} & =\{9,10\},\\ J_{\mathrm{hand}} & =\{7,8,9,10\}, & J_{\mathrm{foot}} & =\{15,16\}. \end{array}\right.$$

**Figure 4:**
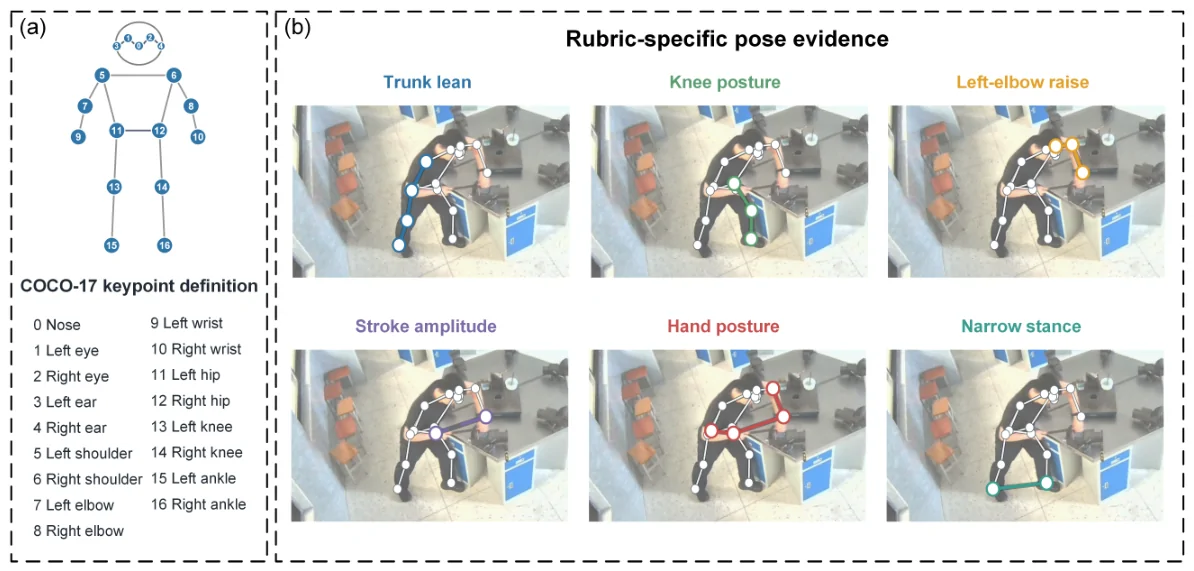
COCO-17 keypoint definition and rubric-specific pose evidence. (**a**) The 17 keypoints and indices used by YOLOv8-Pose; (**b**) rubric-specific keypoint combinations for the six error categories.

The indices follow the COCO-17 convention used by YOLOv8-Pose. Figure 4 shows both the keypoint definition and the body geometry associated with each rubric item.

Using these sets, the category-specific rubric features are calculated as

(6) $$g_{i}^{\mathrm{rubric}}=\Gamma \left({\left\{\psi_{k}\left(P_{i,t};J_{k}^{\mathrm{rubric}}\right)\right\}}_{t=1}^{T}\right),\;k=1,\ldots ,K,$$

where $\psi_{k}$ is the geometric function for error category *k*. Appendix A, Table A2 summarizes the scalar descriptors, aggregation rules, and dimensional contribution of each feature block. The general and rubric-specific features are concatenated into a 438-dimensional raw vector. It comprises 200 general-pose dimensions and 238 rubric-specific dimensions. Within each training fold, this vector is standardized and reduced to 12 principal components. Ten standardized rubric-specific temporal descriptors are retained alongside the PCA components. The final representation is

(7) $$u_{i}=\mathrm{Norm}\;\left([g_{i}^{\mathrm{base}};g_{i}^{\mathrm{rubric}}]\right)\in R^{438},\;x_{i}=\left[{\mathrm{PCA}}_{12}\left(u_{i}\right);\mathrm{Norm}\left(a_{i}^{\mathrm{rubric}}\right)\right]\in R^{22},$$

where $a_{i}^{\mathrm{rubric}}\in R^{10}$ denotes the 10-dimensional directly retained rubric-specific descriptor block. In the present implementation, this block represents the narrow-stance evidence. Because it contains only 10 dimensions, whereas the other feature families contribute substantially more dimensions, its contribution to the dominant covariance directions may be limited when PCA is fitted to the complete 438-dimensional vector. Its criterion-specific signal may therefore be attenuated. We standardize these ten descriptors separately and concatenate them with the 12 PCA components, allowing the encoder to retain explicit narrow-stance evidence while also modeling global pose variation. $x_{i}$ is the final pose representation for video *i*. Standardization and principal component analysis were fitted only on the outer-training participants and then applied to the corresponding outer-test participant.

### 3.5. Error Prototype Diagnosis Module

After obtaining the pose representation, RESC forms an error-semantic diagnostic layer before generating the score. This design follows the instructional process in which deductions are assigned to specific errors and prevents the overall-score supervision from obscuring different error combinations. A lightweight encoder maps $x_{i}$ into a latent space:

(8) $$z_{i}=f_{\theta }\left(x_{i}\right).$$

The encoder $f_{\theta }$ is a lightweight two-layer multilayer perceptron, where θ⊂Θ denotes its trainable parameters. A first linear projection and GELU activation produce a smooth nonlinear response. Layer normalization stabilizes feature scale, and dropout suppresses feature co-adaptation under limited-data training. A second linear layer generates the latent representation $z_{i}$ for prototype matching. To align the latent space with rubric semantics, a prototype vector $c_{k}$ is learned for each error. Each prototype represents the typical evidence center of one error. Its cosine similarity to a sample is

(9) $$e_{i,k}=\cos\left(z_{i},c_{k}\right)=\frac{z_{i}^{T}c_{k}}{{∥z_{i}∥}_{2}{∥c_{k}∥}_{2}}.$$

The similarity is converted into an error logit and probability:

(10) $$\ell_{i,k}=\mathrm{softplus}\left(\alpha_{k}\right)e_{i,k},\;p_{i,k}=\sigma \left(\ell_{i,k}\right).$$

Here, $e_{i,k}$ is the prototype similarity for video *i* and error *k*, and σ(·) denotes the sigmoid function. Applying softplus to the category scale $\alpha_{k}$ keeps the multiplier positive. Unlike independent multilabel heads, the prototypes define shared semantic coordinates against which evidence from different samples can be compared. They also provide a common semantic input to severity estimation and deduction calculation.

### 3.6. Ordered Severity and Adaptive Deduction Gating

Error severity has an ordinal structure from no error to a severe error. Treating levels 0–3 as unrelated classes would discard this ordering, where 0 indicates an absent or negligible error and 3 indicates a major effect on action quality. RESC therefore uses a cumulative-link model based on $\ell_{i,k}$:

(11) $$P\left(s_{i,k}\geq m\right)=\sigma \;\left(\gamma_{k,m}\left(\ell_{i,k}-\tau_{k,m}\right)\right),\;m=1,2,3,$$

(12) $${\hat{s}}_{i,k}=\sum_{m=1}^{3}P\left(s_{i,k}\geq m\right).$$

Here, $s_{i,k}$ is the observed severity, $\gamma_{k,m}$ is a nonnegative slope constrained by softplus, and $\tau_{k,m}$ is a category-specific threshold. The three thresholds are sorted during the forward pass so that $\tau_{k,1}\leq \tau_{k,2}\leq \tau_{k,3}$. Equation (12) converts the three cumulative probabilities into the expected severity ${\hat{s}}_{i,k}$. This preserves ordinal information while producing a continuous quantity for the deduction branch. Ordinal loss is evaluated only where valid severity labels are available.

The contribution of a predicted severity depends on the sample representation and its category-specific diagnostic evidence. RESC models this variation with a learnable adaptive deduction gate. Let $e_{i}=\left[e_{i,1},\ldots ,e_{i,K}\right]$ denote the prototype-similarity vector. The six gate values are computed as

(13) $${\hat{r}}_{i}=\sigma \;\left(h_{\phi }\;\left([z_{i};e_{i}]\right)\right)\in {\left(0,1\right)}^{K},$$

where $h_{\phi }$ is a two-layer gate network with dimensions 134–64–6 and a GELU activation between the two linear transformations. The sigmoid function maps each component to a sample- and item-specific gate ${\hat{r}}_{i,k}$. Its parameters ϕ⊂Θ are optimized jointly with the encoder, error prototypes, severity branch, and scoring branch through the total training objective.

### 3.7. Nonnegative Deductions and Conditional Error–Score Consistency

Vocational skill assessment has a clear directional constraint: all else being equal, greater error severity should reduce the final score. RESC generates an interpretable score through an explicit deduction branch. The model-derived deduction contribution for error *k* is

(14) $${\hat{d}}_{i,k}=\mathrm{softplus}\left(w_{k}\right){\hat{r}}_{i,k}{\hat{s}}_{i,k},$$

where $\mathrm{softplus}(w_{k})$ is a nonnegative deduction weight. Its initial value is obtained by fitting the observed severity of each error to the total deduction $100-y_{i}$ using nonnegative linear regression within the training fold, and it is further updated during joint training. The final score is calculated by subtracting all itemized deductions from the full-score reference:

(15) $${\hat{y}}_{i}=\mathrm{clip}\;\left(100-\mathrm{softplus}\left(b\right)-\sum_{k=1}^{K}{\hat{d}}_{i,k},0,100\right).$$

The nonnegative bias softplus(b) is initialized from the intercept of the same training-fold regression and updated during training. It calibrates the baseline contribution not covered by the six errors, while clip constrains predictions to 0–100. The bias is reported separately from the six rubric deductions as a residual score-calibration term. For fixed nonnegative weights and gate values, the item deduction is nondecreasing in expected severity; for fixed severity, it is nondecreasing in the gate value. These conditional directional constraints define the local behavior of the deduction equation. Section 4.3.3 evaluates the complete learned mapping when prototype evidence, severity, and gate values change jointly. The branch exposes an auditable model-derived contribution for every error.

### 3.8. Training Objective and Inference

During training, RESC is jointly supervised by instructor scores, quality levels, error categories, and error severities. The total objective combines score regression, error classification, ordinal severity, quality-level classification, score consistency, and deduction-weight regularization:

(16) $$L=\lambda_{s}L_{\mathrm{score}}+\lambda_{e}L_{\mathrm{err}}+\lambda_{o}L_{\mathrm{ord}}+\lambda_{l}L_{\mathrm{level}}+\lambda_{c}L_{\mathrm{cons}}+\lambda_{w}L_{w}.$$

$L_{\mathrm{score}}$ combines Smooth L1 supervision of the deduction-chain score with a lower-weight Smooth L1 term from an auxiliary direct-scoring head. The auxiliary head concatenates $z_{i}$, the six prototype similarities, and the normalized expected severities, and maps them through a 128-unit GELU–LayerNorm–Dropout block and a sigmoid output to the interval 0–100. During inference, the deduction-chain branch provides the final score, while the direct head contributes auxiliary supervision during training. $L_{\mathrm{err}}$ is binary cross-entropy weighted by the positive-class proportion in each training fold. $L_{\mathrm{ord}}$ combines binary cross-entropy on cumulative probabilities with the absolute error of expected severity and masks missing severity labels. $L_{\mathrm{level}}$ is cross-entropy for the quality level. $L_{\mathrm{cons}}$ constrains pairwise rankings between predicted and instructor scores and prevents scores from increasing as the predicted total severity burden grows. Finally, $L_{w}$ prevents learned nonnegative deduction weights from drifting excessively from their training-fold initialization. Their explicit definitions are

(17) $$L_{\mathrm{cons}}=\frac{1}{|P_{y}|}\sum_{\left(i,j\right)\in P_{y}}\mathrm{softplus}\;\left[-\frac{\mathrm{sgn}\left(y_{i}-y_{j}\right)\left({\hat{y}}_{i}-{\hat{y}}_{j}\right)}{5}\right]+\frac{0.35}{|P_{s}|}\sum_{\left(i,j\right)\in P_{s}}\mathrm{softplus}\;\left[\frac{\mathrm{sgn}\left(S_{i}-S_{j}\right)\left({\hat{y}}_{i}-{\hat{y}}_{j}\right)}{5}\right]$$

(18) $$L_{w}=\frac{1}{K}\sum_{k=1}^{K}\mathrm{SmoothL1}\;\left(\mathrm{softplus}\left(w_{k}\right),w_{k}^{\left(0\right)}\right),$$

where $S_{i}=\sum_{k}{\hat{s}}_{i,k}$, $P_{y}=\{\left(i,j\right):|y_{i}-y_{j}\left|>1\right\}$, and $P_{s}=\{\left(i,j\right):|S_{i}-S_{j}\left|>0.25\right\}$. The value $w_{k}^{\left(0\right)}$ is the nonnegative training-fold initialization described above. Empty valid-pair sets contribute zero to the corresponding term. The coefficients $\lambda_{s}$ through $\lambda_{w}$ control the contribution of each objective. Together, these losses supervise different levels of the scoring chain so that the model learns overall quality, error semantics, and ordinal severity jointly.

## 4. Experiments and Results

### 4.1. Training Environment and Evaluation Metrics

The models were implemented in PyTorch 2.5.1 with CUDA 12.1 and trained on an NVIDIA GeForce RTX 4090 Laptop GPU with 24 GB of memory. Cross-subject performance was evaluated using outer LOSO cross-validation, with one participant reserved for testing in each fold. Among the remaining participants, one complete participant formed the inner validation set for early stopping and threshold selection, while the others were used for gradient-based optimization. All preprocessing statistics, initialization quantities, and model-selection operations were confined to the outer-training fold. Binary error thresholds maximized balanced accuracy on inner-validation predictions; when a category contained only one validation class, the corresponding inner-training threshold was used. The three severity cut points minimized discrete severity MAE over values from 0.2 to 2.8 at intervals of 0.1. All thresholds were fixed before outer-test evaluation. The fixed architecture, optimization settings, early-stopping objective, and loss coefficients are summarized in Appendix A, Table A1.

The inner-validation participant was selected deterministically within each outer-training fold. Participants contributing at least three videos were considered first, and the participant whose video count was closest to 10% of the outer-training videos was selected; ties were resolved by participant identifier. If no participant met this condition, the last identifier in sorted order was used. The selection rule depends only on participant identifiers and video counts and does not use scores, error labels, or outer-test predictions. The PCA output dimension (12), the ten retained rubric-specific descriptors, the encoder and gate widths, and the loss coefficients were fixed as model-design settings before the reported outer LOSO runs and were held constant in all 25 folds. Standardization and PCA transformation parameters were fitted separately within each outer-training fold. These design settings remained fixed throughout the outer LOSO evaluation and were determined independently of the pooled outer-test results.

Model performance was evaluated using four complementary metrics, whose definitions are given in Equations (19)–(22). The Spearman rank correlation coefficient (SRCC) measures rank-order agreement between predicted and instructor scores, whereas the Pearson linear correlation coefficient (PLCC) measures their linear agreement. Mean absolute error (MAE) reports the average magnitude of score errors, while root mean square error (RMSE) gives greater weight to large deviations.

(19) $$\mathrm{SRCC}=1-\frac{6\sum_{i=1}^{N}d_{i}^{2}}{N(N^{2}-1)},$$

(20) $$\mathrm{PLCC}=\frac{\mathrm{Cov}(y,\hat{y})}{\sigma_{y}\sigma_{\hat{y}}},$$

(21) $$\mathrm{MAE}=\frac{1}{N}\sum_{i=1}^{N}\left|y_{i}-{\hat{y}}_{i}\right|,$$

(22) $$\mathrm{RMSE}=\sqrt{\frac{1}{N}\sum_{i=1}^{N}{\left(y_{i}-{\hat{y}}_{i}\right)}^{2}}.$$

In Equations (19)–(22), *N* is the total number of test videos, while $y_{i}$ and ${\hat{y}}_{i}$ denote the instructor-assigned and predicted scores for video *i*, respectively. In Equation (19), $d_{i}$ is the difference between the ranks of $y_{i}$ and ${\hat{y}}_{i}$. In Equation (20), *y* and $\hat{y}$ are the instructor-assigned and predicted score vectors, $\mathrm{Cov}(y,\hat{y})$ is their covariance, and $\sigma_{y}$ and $\sigma_{\hat{y}}$ are their standard deviations. The four metrics were calculated by pooling the predictions from all 25 outer-test folds. All comparison and ablation experiments report these four metrics.

### 4.2. Comparison with Representative Baselines

To evaluate cross-subject generalization across different assessment paradigms, we considered three groups of baselines: elastic alignment based on global sequence distance, feature-learning methods based on video or skeleton representations, and structured methods that explicitly incorporate rubrics or scoring rules. Because Fitter-AQA is newly constructed, every reported method was trained and tested on this dataset. For methods whose published implementation could not be transferred directly to Fitter-AQA, we constructed principle-based adaptations that preserve the core mechanisms described in the original publications and align their inputs and outputs with Fitter-AQA. These implementations are labelled “adapted”. All baselines used the same outer LOSO partitions as RESC, and their preprocessing, parameter selection, and model selection were restricted to the corresponding outer-training participants. The outer-test participant in each fold was used only for final evaluation. Table 2 summarizes the core interpretation of each structured method, the modification made for comparison with RESC, and its training configuration.

#### 4.2.1. Elastic-Alignment Quality Assessment

Table 3 reports the elastic sequence-alignment results. The variants differ in their strengths for absolute calibration and rank correlation, indicating that a single global distance does not optimize both objectives simultaneously. RESC produced the lowest pooled errors and highest pooled correlations in this comparison. Relative to the strongest alignment baseline for each corresponding metric, MAE decreased from 7.730 to 4.450 and SRCC increased from 0.652 to 0.852. These pooled point estimates favor structured error evidence over whole-sequence similarity for cross-subject quality discrimination.

Elastic alignment can absorb variation in action speed and phase duration, but its prediction remains dominated by cumulative distance over the full sequence. In filing, quality differences often occur as brief and local postural deviations. These cues can be diluted by normal periodic motion during global matching. RESC instead organizes local geometric evidence by rubric item and explicitly models the direction from error to deduction, retaining scoring resolution even when participants follow similar overall motion patterns.

#### 4.2.2. End-to-End Feature-Learning Methods

To compare RESC with end-to-end feature learning, we evaluated representative AQA baselines based on skeleton topology, score distributions, relative regression, and temporal parsing. Table 4 shows that feature-learning methods generally outperform elastic-alignment baselines, but remain limited in both error and correlation under strict cross-subject evaluation. In the pooled outer-test predictions, RESC reduced MAE and RMSE by 33.3% and 37.5%, respectively, relative to USDL, while increasing PLCC by 0.164 and SRCC by 0.182. These values describe pooled point-estimate differences; their participant-level uncertainty is reported in Appendix A, Table A3.

The difference reflects supervision granularity and task structure. Global representation learning captures motion rhythm and overall motion patterns, and distributional or ranking supervision improves uncertainty estimates and relative order. Under limited-sample cross-subject evaluation, however, video-level mappings may still exploit non-quality cues such as body shape and clothing. RESC grounds supervision in local posture evidence specified by the rubric. This representation directs learning toward action errors, reduces reliance on subject-specific variation unrelated to the rubric, and is consistent with the improved pooled cross-subject results.

#### 4.2.3. Structured Scoring Methods

To determine whether rubrics and scoring rules improve small-sample skill assessment, structured scoring methods were evaluated separately in Table 5. These methods decompose the overall score into action steps or technical elements with explicit intermediate semantics. Because their semantic structures differ, each method was adapted while preserving its central mechanism.

Methods that convert local pose evidence into error symbols and score them using explicit rules adapted more effectively to this task than the preceding two groups. This finding indicates that structured priors are most useful when linked to concrete instructional errors. Relative to the strongest structured baseline, RESC reduced MAE by 33.7% and improved PLCC and SRCC by 0.100 and 0.182, respectively. Decomposing an action into steps or technical elements alone does not ensure stable cross-subject scoring because intermediate diagnostic errors can propagate during score aggregation. RESC jointly optimizes error prototypes, ordinal severity, and monotonic deduction. Local evidence therefore supports both diagnosis and final-score constraints within one consistent assessment chain.

We additionally compared RESC with USDL and NS-AQA-adapted using 10,000 paired participant-cluster bootstrap resamples (Appendix A, Table A3). All point differences favored RESC. For NS-AQA-adapted, the 95% intervals excluded zero for all four metrics. For USDL, the intervals crossed zero, indicating that the observed pooled advantage over this feature-learning baseline does not constitute a formal significance claim at the participant level.

#### 4.2.4. Controlled Same-Input Comparison and Statistical Stability

To separate the effect of the input representation from that of the structured scoring mechanism, we added three controlled comparisons. Ridge regression and a two-layer multilayer perceptron used the same 438-dimensional pose and rubric feature set, the same fold-fitted standardization and PCA, and the same overall-score labels as RESC. The auxiliary direct-score head additionally shared the RESC encoder and multi-objective training supervision and mapped the shared representation directly to the score. All four outputs were evaluated using the same outer LOSO splits. To quantify subject-level variability, 95% confidence intervals were obtained from 10,000 bootstrap resamples using the outer-test participant as the clustering unit. Detailed results are presented in Table 6.

Under identical pose evidence, Full RESC achieved the lowest MAE and RMSE and the highest PLCC and SRCC among the four controlled models. It also improved all four metrics over the auxiliary direct-score head, which shared the same representation and multi-task supervision, supporting the additional contribution of deduction-chain aggregation. Across 10,000 participant-cluster resamples, the PLCC and SRCC intervals remained within 0.837–0.944 and 0.665–0.887, respectively, indicating stable score agreement under subject-level variation. Across the 25 held-out participants, the subject-level MAE had a median of 4.60 and an interquartile range of 3.21–7.93.

### 4.3. Ablation Studies

#### 4.3.1. Feature-Evidence Ablation

To evaluate the independent and complementary roles of general pose statistics and rubric geometry, we trained RESC with each evidence source separately and with their fusion. Table 7 shows that general pose statistics favor absolute score calibration, producing an MAE of 6.749 when used alone. Rubric geometry preserves quality order more effectively, reaching an SRCC of 0.721. Their fusion reduced MAE to 4.450 and increased SRCC to 0.852. Overall motion context and local rubric evidence are therefore complementary, supporting the dual-evidence design of RESC.

General statistics summarize motion stability and individual movement scale, whereas rubric geometry directly represents scoring evidence. The former supplies the global context needed for score calibration, and the latter establishes the evaluation direction for fine-grained quality differences. Their combination yields the strongest overall performance.

#### 4.3.2. Component Ablation

To determine how each component contributes to scoring and structural consistency, we removed one key design at a time and retrained the model. Table 8 shows that error prototypes have the largest effect: their removal increased MAE to 7.546 and reduced SRCC to 0.645. Removing ordinal severity or adaptive deduction gating also degraded overall performance. Without monotonicity or consistency constraints, individual metrics approached the full model, but scoring error and rank correlation did not remain strong simultaneously. The complete RESC consequently provides the best joint behavior.

Error prototypes provide semantic anchors for local postural deviations. Ordinal severity and adaptive deduction gating then determine sample-specific deduction magnitude, while monotonicity and consistency stabilize the direction between error burden and final score. Their combined effect allows RESC to balance numerical accuracy, ranking stability, and deduction logic.

#### 4.3.3. Counterfactual Directional-Consistency Analysis

To empirically examine the directional behavior of the complete deduction mapping, we performed a counterfactual stress test on the outer-LOSO predictions. For one rubric item at a time, its prototype evidence was varied over the observed value ±0.50 while the shared representation was held fixed; the corresponding severity and adaptive deduction gate were then recomputed. For every pair of adjacent intervention levels, we tested whether stronger error evidence produced a nondecreasing item deduction and a nonincreasing final score. Detailed statistics for the counterfactual stress test are summarized in Table 9.

Across all 29,309 adjacent interventions, increasing the prototype evidence consistently increased the corresponding item deduction. The final score followed the expected direction in 99.22% of the comparisons. The remaining 228 reversals were confined to narrow stance and had a maximum magnitude of only 0.0016 points. All comparisons satisfied directional consistency under a numerical tolerance of 0.01 points.

### 4.4. Error Diagnosis

To evaluate whether RESC identifies specific rubric errors under cross-subject conditions, we performed category-level analysis using predictions from the outer LOSO test folds. A video may contain several errors, so each rubric item was treated as an independent binary task. F1 balances error recall and prediction precision at the selected threshold. Average precision (AP) evaluates confidence ranking over all decision thresholds. Balanced accuracy weights error and no-error samples equally, reducing the influence of class imbalance. Together, the metrics assess fixed-threshold discrimination, confidence ordering, and class-balanced recognition.

**Figure 5:**
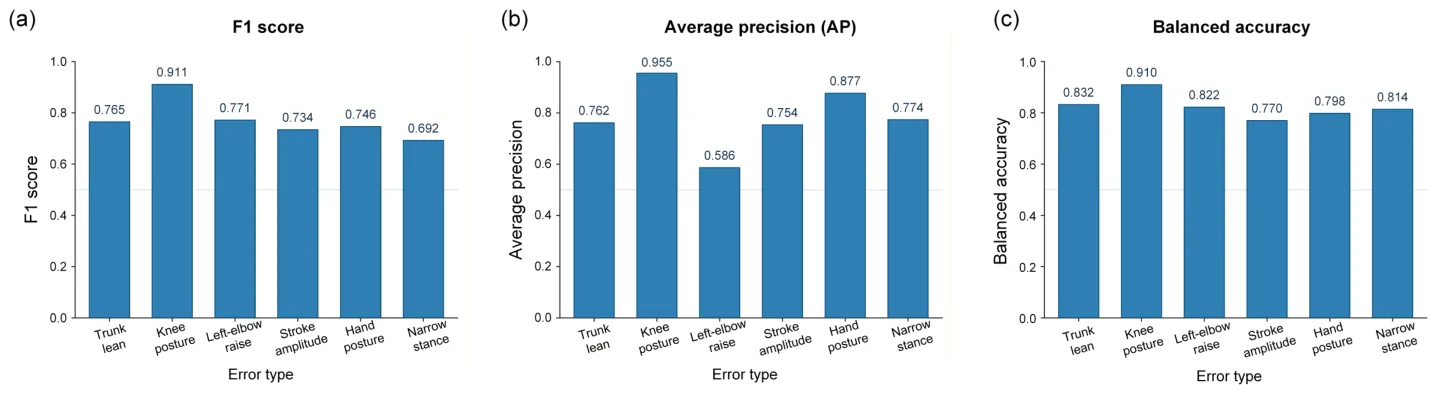
Category-level recognition results for the six rubric errors: (**a**) F1; (**b**) average precision (AP); and (**c**) balanced accuracy. All metrics were calculated from outer-LOSO test predictions for 134 videos from 25 participants. The vertical scale is 0–1.

As shown in Figure 5, F1 ranged from 0.692 to 0.911, with a Macro-F1 of 0.770. RESC therefore maintained a useful balance between detecting true errors and rejecting no-error cases. Knee posture exceeded 0.90 on all three metrics, indicating stable pose evidence and a clear class boundary. Left-elbow raise achieved strong F1 and balanced accuracy but a lower AP, showing that thresholded discrimination was effective even though confidence ranking remained less stable. Narrow stance had the lowest F1, while its balanced accuracy remained 0.814; its main difficulty was therefore the precision–recall trade-off rather than a failure to separate error and no-error samples. The complementary results show that score-relevant pose evidence can be converted into diagnoses with explicit rubric semantics.

**Figure 6:**
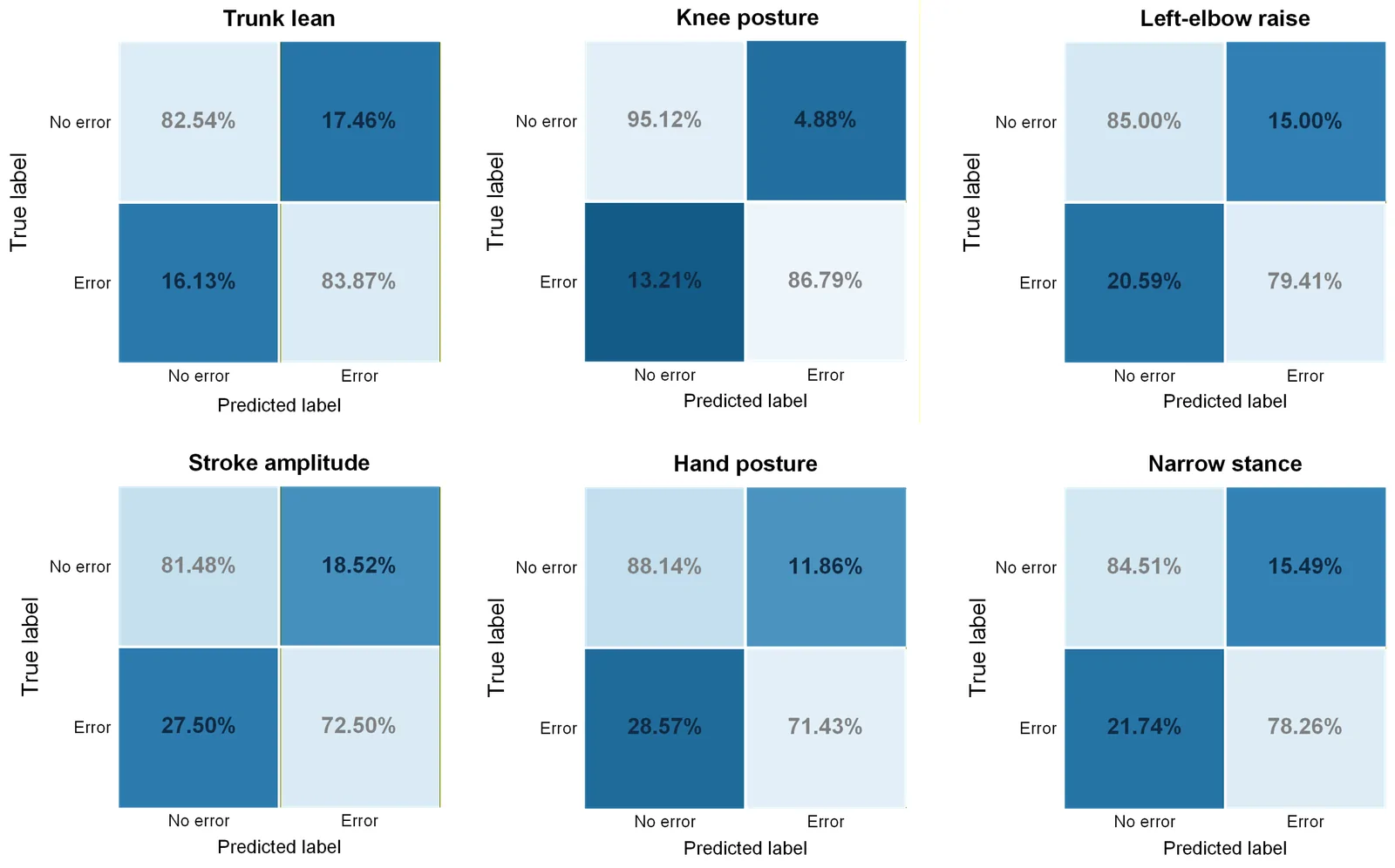
One-vs.-rest row-normalized confusion matrices for the six rubric errors. Rows denote observed labels and columns denote predicted labels. The upper-left cell in each panel is the correctly rejected error rate, the lower-right cell is the correctly detected error rate, and every row sums to 100%.

Figure 6 provides a direct view of detection behavior. Specificity ranged from 81.48% to 95.12%, and recall ranged from 71.43% to 86.79%. Thus, every category correctly rejected at least 81.48% of no-error samples and detected at least 71.43% of true errors. Specificity exceeded recall for most categories, indicating a relatively conservative decision policy in which the remaining errors primarily arise from missed detections. Together with F1 and balanced accuracy, these matrices show that the diagnostic results are not an artifact of the majority class and can support itemized deductions.

#### Ordinal Severity Evaluation

**Figure 7:**
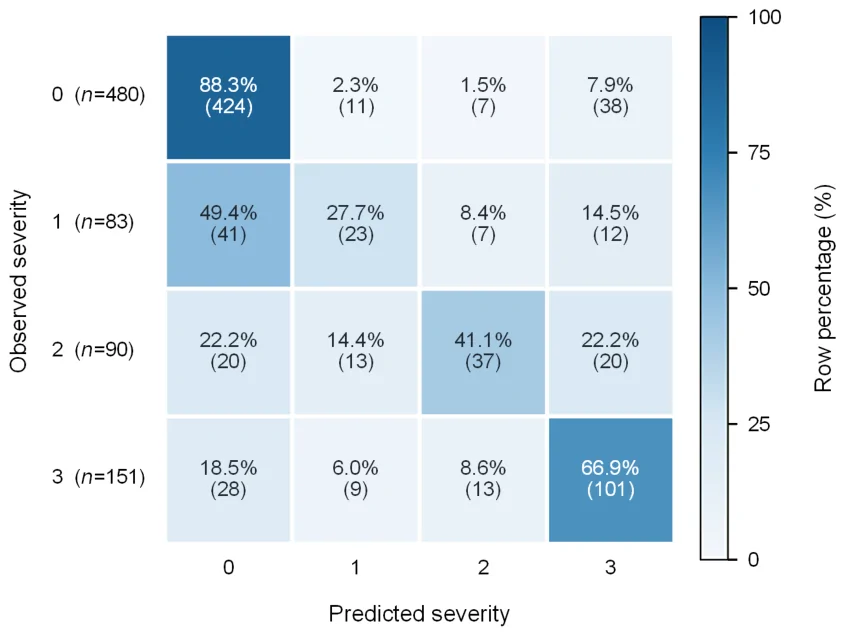
Row-normalized confusion matrix for ordinal severity prediction. The analysis contains 804 rubric-item labels from 134 outer-LOSO test videos. Rows denote observed levels and columns denote predicted levels; each cell reports its row percentage and sample count.

**Figure 8:**
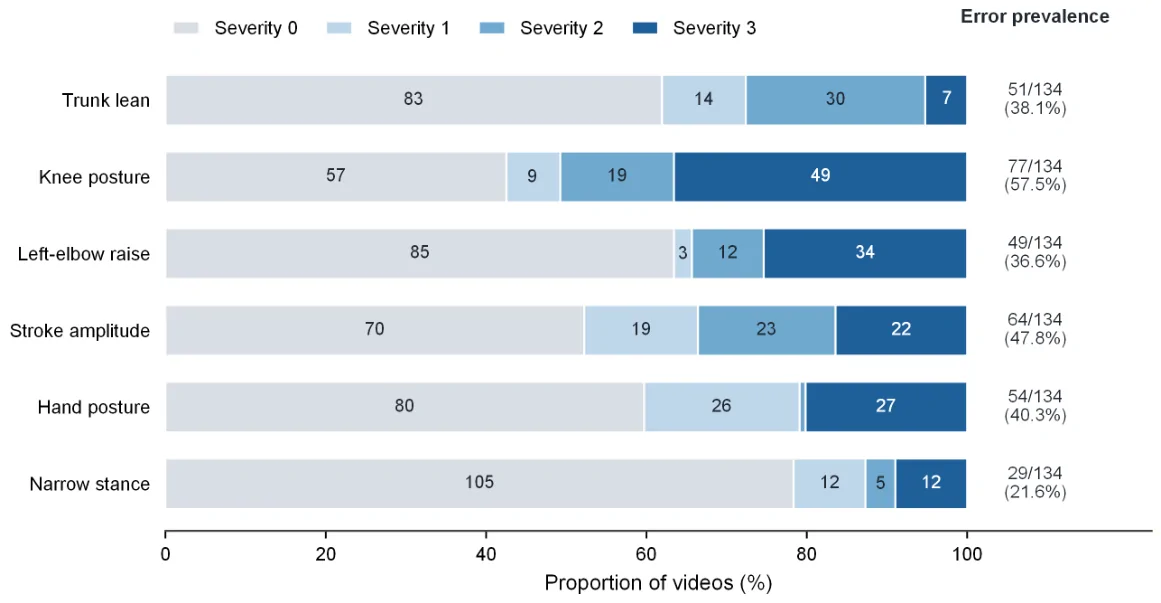
Severity-level distribution and error prevalence for the six rubric categories. Each horizontal bar contains 134 videos and is divided by observed severity level. Sample counts are displayed within segments where space permits. Error prevalence is the number and proportion of videos with severity levels 1–3.

The ordinal branch was evaluated over all 804 rubric-item labels obtained by pooling the six error categories across the 134 outer-LOSO test videos. Discrete-level MAE and exact accuracy evaluate the decoded levels 0–3. Within-one-level accuracy measures the proportion of predictions differing from the observed level by at most one grade. Quadratic-weighted Cohen’s kappa (QWK) additionally accounts for the ordered distance between grades. Table 10 summarizes the ordinal metrics, while Figure 7 and Figure 8 present the confusion pattern and label distribution, respectively.

RESC achieved an exact ordinal accuracy of 72.76%, a within-one-level accuracy of 85.82%, and a QWK of 0.631. Figure 7 shows that levels 0 and 3 were identified more consistently, whereas levels 1 and 2 were more frequently assigned to the endpoints. Figure 8 shows that the two intermediate levels were substantially less represented than level 0 in the pooled labels. This imbalance limits the training evidence available for learning adjacent severity boundaries and contributes to the lower stability of intermediate-grade predictions. The model therefore captured the broad progression from absent to severe error more reliably than the distinction between adjacent intermediate grades.

### 4.5. Visualization of Interpretable Scoring

**Figure 9:**
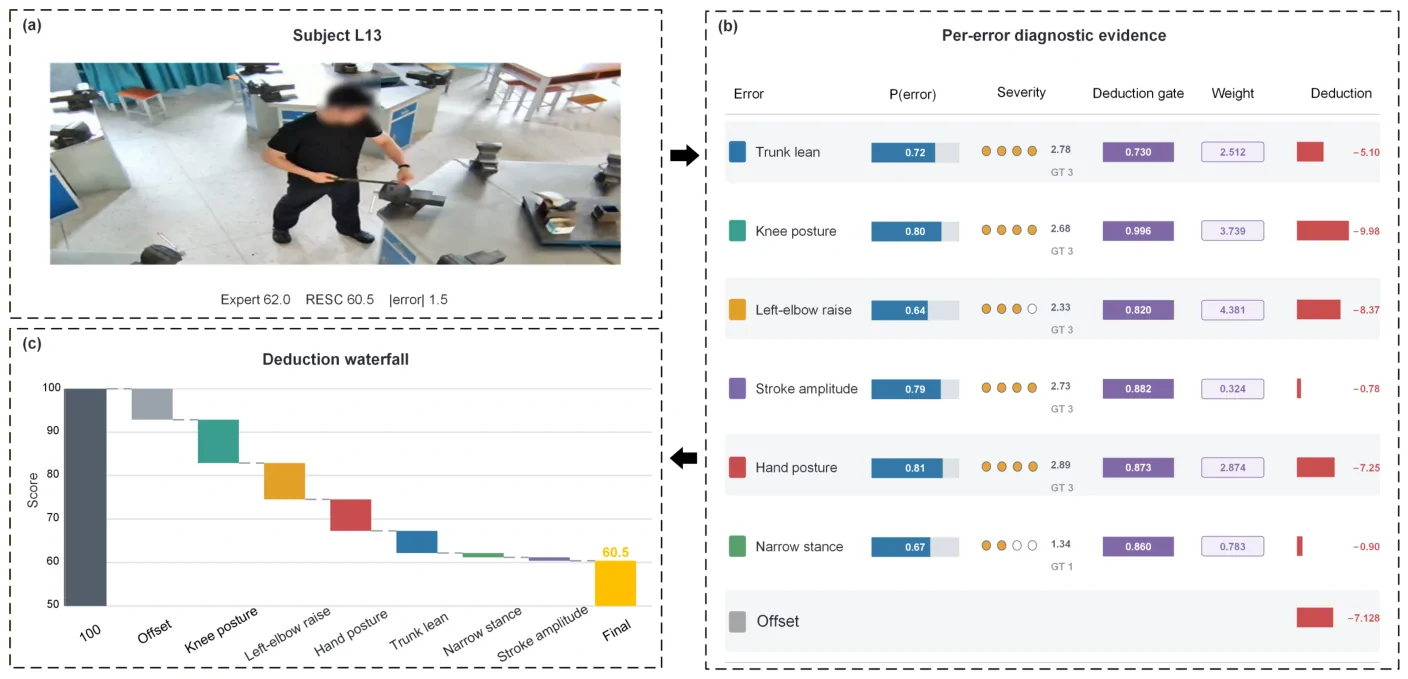
Example of interpretable RESC scoring for test participant L13. (**a**) Input video and scoring result; (**b**) diagnostic evidence and model-derived deduction contribution for each rubric item; and (**c**) waterfall chart that derives the final score from the 100-point reference.

To illustrate the auditability of RESC’s scoring process, we visualized its complete scoring process. Figure 9 uses participant L13 from an outer LOSO test fold and presents both the instructor score and model prediction. Diagnostic evidence for each rubric item is converted into a model-derived deduction contribution, and a waterfall chart aggregates those contributions into the final score. The instructor score for this sample was 62.0, whereas RESC predicted 60.5, giving an absolute error of 1.5 points. Starting from 100, the residual calibration term accounted for 7.128 points of the predicted deduction, and the six rubric errors deducted a further 32.28 points. Knee posture, left-elbow raise, and hand posture were the main sources of score loss, contributing 9.88, 8.37, and 7.25 points, respectively. Stroke amplitude and narrow stance each contributed less than one point.

By converting a score into auditable rubric evidence, the visualization allows an instructor to identify which errors contributed to a low predicted score and prioritize corrective review. Each displayed deduction is a model-derived component linked to a specific rubric item and estimated under overall-score, error-category, and severity supervision. Figure 9 therefore demonstrates the auditability of the model’s score decomposition. The figure establishes traceability within the model and provides a basis for future instructor studies of the pedagogical correctness and practical usefulness of these explanations.

## 5. Discussion

This study shows that an assessment rubric can provide an effective prior linking deep pose representations to vocational skill evaluation. Under strict cross-subject evaluation with 25 participants and 134 videos, RESC reported the lowest pooled errors and highest pooled correlations among the evaluated methods. The observed differences are consistent with a narrower assessment space: pose representations reduce appearance and scene variation, while rubric-aligned evidence further restricts learning to body relations directly associated with operating standards. The model is therefore less dependent on discovering scoring rules from data alone, which is especially valuable for the limited datasets typical of authentic industrial training.

The experiments further demonstrate the traceability of the scoring process. Category-level diagnoses and deduction visualizations connect model outputs to rubric items and decompose the final score into auditable, model-derived contributions. Compared with explanations that only highlight regions or action segments, these outputs provide an explicit representation that can be inspected against the scoring rubric. The present evidence supports rubric alignment and model auditability. Claims about instructional correctness, feedback effectiveness, or user benefit require evaluation by instructors and trainees.

The results further suggest that vocational-skill AQA need not follow the same modeling paradigm as large-scale sports video assessment. When the overall procedure is stable and quality differences are concentrated in local operating standards, combining deep pose estimation with domain rules can preserve critical quality information while controlling model complexity. This finding is consistent with recent pose-aware, fine-grained, and rubric-driven AQA studies that emphasize human-centered evidence and structured explanations [22,25,40,41,42]. Its applicability still depends on the clarity of the scoring rules and on whether the relevant operating evidence can be reliably observed from visual pose.

The current framework relies on body pose and therefore primarily captures gross-body kinematics. COCO-17 keypoints provide reliable evidence for posture and limb coordination but offer limited representation of fine hand–tool coordination, tool orientation, and tool–workpiece contact. Dimensional accuracy and surface quality are properties of the finished workpiece and require direct measurement. RESC therefore evaluates the pose-observable component of filing performance. A more comprehensive assessment should combine body pose with hand and tool tracking, contact-aware visual evidence, and direct workpiece-quality measurements.

The ordinal severity analysis also identifies a limitation in fine-grained grading. The separation of severity levels 1 and 2 was less stable than that of the endpoint levels. Intermediate-grade samples were relatively sparse, and adjacent levels were distinguished according to deviation magnitude, persistence, and functional influence, whose visual boundaries may overlap in pose-based observations. The observed confusion therefore reflects both the current ordinal branch’s limited resolution between adjacent grades and the intrinsic difficulty of four-level severity assessment under a small and imbalanced dataset. The present evidence supports error-category recognition and broad severity progression, while precise differentiation between adjacent intermediate grades remains limited.

The current evaluation is also limited to one filing task recorded in a single vocational training workshop. The unequal number of videos contributed by individual participants reflects the natural collection process. Because video-level camera and workstation identifiers were not retained, camera- or workstation-related confounding cannot be excluded, and acquisition-unit sensitivity cannot be estimated from the present dataset. Generalization to different workshops, acquisition layouts, trainee cohorts, and other vocational operations requires further evaluation.

## 6. Future Work

Future research will integrate operation-process assessment with evaluation of the manufactured workpiece. RESC currently assesses action quality mainly through human pose and rubric errors, whereas the goals of fitter training also include dimensional accuracy, surface quality, and form error. Hand keypoints, tool and workpiece detection, and direct workpiece measurements could complement the current pose evidence. Linking action videos with workpiece measurements could reveal relationships between operating errors and manufacturing defects. Such a system would provide a more complete assessment of procedural correctness, process stability, and output quality, while explaining how specific motion deviations affect the finished workpiece.

Future work will expand Fitter-AQA by including more participants, training workshops, acquisition conditions, and vocational tasks. Targeted collection of the underrepresented intermediate severity levels will improve label balance, support more reliable ordinal learning, and enable broader evaluation of cross-scenario generalization.

Reducing instructor annotation cost is another important direction. The current training process uses supervision at several semantic levels, which limits rapid dataset expansion. Semi-supervised learning, active learning, and pseudo-labeling could route the most informative or least confident samples to instructors and use a small amount of detailed annotation to exploit larger collections of unlabeled video. These strategies may reduce repetitive labeling while preserving scoring reliability. They may also make it easier to transfer rubric-guided assessment to other vocational trades, where the evidence definitions and scoring rules differ but the need for interpretable cross-subject evaluation remains. A future instructor study will separately assess the pedagogical correctness, clarity, and practical usefulness of the model-derived diagnoses and deduction decomposition.

## Figures and Tables

**Table 1:** Operational definitions, pose evidence, and severity protocol for the six Fitter-AQA rubric errors.

(**a**) Error Definitions and Pose Evidence		
**Error category**	**Instructional definition**	**Pose evidence**
Trunk lean	Insufficient or excessive forward trunk lean, or pronounced trunk sway during filing.	Angle and temporal variation of the right shoulder–hip–knee–ankle alignment.
Knee posture	Knee hyperextension, insufficient or excessive flexion, or unstable lower-body support.	Left hip–knee–ankle angle and its temporal variation.
Left-elbow raise	Noncompliant left-elbow height or failure to maintain a stable elbow position.	Relative height and joint angle of the left shoulder, elbow, and wrist.
Stroke amplitude	The filing stroke is too short and the effective reciprocating range is insufficient.	Horizontal trajectory lengths of both wrists.
Hand posture	Grip, force application, or coordination between the two hands does not comply with the operating requirements.	Elbow–wrist configuration, inter-wrist distance, and wrist-position proxy features.
Narrow stance	Foot spacing is too narrow to provide a stable base for standing and force application.	Inter-ankle distance, its body-scale-normalized value, and temporal variation.
(**b**) Common ordinal severity scale		
**Severity level**	**Operational definition**	
0—Absent	No observable violation of the corresponding rubric criterion.	
1—Mild	A small or occasional deviation with limited influence on execution.	
2—Moderate	A clear or recurrent deviation that affects stability or compliance.	
3—Severe	A pronounced or persistent deviation that substantially affects action quality.	

**Table 2:** Core mechanisms, Fitter-AQA adaptations, and training settings of the structured AQA baselines. RICA^2^-adapted, RCS-adapted, and IRIS-adapted used a common 48×1024 I3D sequence with training-fold standardization and PCA(64), whereas NS-AQA-adapted used six rubric-specific pose groups. All methods used the same 134 videos and outer LOSO splits.

Method	Core Interpretation	Adaptation to Fitter-AQA	Training Settings
RICA^2^-adapted	Models rubric criteria as graph nodes and propagates criterion evidence to an uncertainty-aware score.	The graph was rebuilt with six error nodes and one score root. Outputs were aligned to error evidence, severity, and continuous score using the common I3D input.	One-layer Transformer, hidden dimension 64, four heads, dropout 0.15; AdamW, learning rate 0.001, weight decay 0.002; maximum 260 epochs, patience 35; 24 Monte Carlo samples; seeds 17, 42, and 73.
RCS-adapted	Uses text-defined rubric queries to extract criterion evidence and aggregate criterion scores.	The six fitter rubric descriptions replaced the original task criteria, and temporal attention learned criterion-relevant segments directly from video-level supervision.	Same temporal backbone and optimizer as RICA^2^-adapted; maximum 260 epochs, patience 35; attention regularization 0.02; seeds 17, 42, and 73.
IRIS-adapted	Uses rubric-item queries to obtain item-level evidence and subscores before global scoring.	Six video-level error items replaced action-segment criteria, and weak item localization learned item-relevant evidence from video-level labels.	Same temporal backbone and optimizer as RICA^2^-adapted; maximum 260 epochs, patience 35; attention regularization 0.02; seeds 17, 42, and 73.
NS-AQA-adapted	Represents criteria as neural symbols and combines ordered severity states through nonnegative rules.	Six rubric-specific pose groups generated four-level severity symbols and rule-based score deductions.	Per-criterion MLP, hidden dimension 32, dropout 0.15; AdamW, learning rate 0.002, weight decay 0.002; maximum 500 epochs, patience 50; seeds 17, 42, and 73.

**Table 3:** Cross-subject scoring performance of RESC and elastic sequence-alignment methods. Down and up arrows indicate that lower and higher values are better, respectively. The best value in each column is shown in bold.

Method	MAE ↓	RMSE ↓	PLCC ↑	SRCC ↑
CDTW-KRR [48]	8.991	11.951	0.488	0.418
DDTW-KRR [49]	8.506	10.286	0.692	0.652
WDTW-KRR [50]	9.161	12.220	0.463	0.407
ShapeDTW-KRR [51]	9.100	12.110	0.473	0.407
Soft-DTW-KRR [52]	8.362	11.282	0.544	0.459
TWED-KRR [53]	7.730	10.158	0.666	0.533
ADTW-KRR [54]	9.070	12.102	0.474	0.413
**RESC**	**4.450**	**5.870**	**0.902**	**0.852**

**Table 4:** Cross-subject scoring performance of RESC and representative feature-learning AQA methods. Skeleton networks use the same skeleton-sequence input, while video-based AQA methods use a common I3D temporal representation. Down and up arrows indicate that lower and higher values are better, respectively. The best value in each column is shown in bold.

Method	MAE ↓	RMSE ↓	PLCC ↑	SRCC ↑
ST-GCN [4]	9.178	11.783	0.491	0.467
2s-AGCN [55]	9.011	11.807	0.550	0.498
USDL [23]	6.674	9.395	0.738	0.670
CoRe [6]	8.352	10.468	0.630	0.632
CTR-GCN [56]	9.371	11.938	0.535	0.482
TPT [21]	8.832	11.886	0.567	0.586
**RESC**	**4.450**	**5.870**	**0.902**	**0.852**

**Table 5:** Cross-subject scoring performance of RESC and rubric- or rule-guided AQA methods. The adapted RICA^2^, RCS, and IRIS variants use a common I3D temporal representation, while NS-AQA-adapted uses the six groups of rubric-aligned pose geometry. Down and up arrows indicate that lower and higher values are better, respectively. The best value in each column is shown in bold.

Method	MAE ↓	RMSE ↓	PLCC ↑	SRCC ↑
IRIS-adapted [41]	8.627	11.199	0.563	0.577
RICA^2^-adapted [42]	8.074	10.830	0.616	0.607
NS-AQA-adapted [44]	6.709	8.059	0.802	0.670
RCS-adapted [43]	8.569	10.868	0.591	0.584
**RESC**	**4.450**	**5.870**	**0.902**	**0.852**

**Table 6:** Controlled scoring comparison under identical pose evidence and outer LOSO splits. Values in parentheses are participant-cluster bootstrap 95% confidence intervals. Arrows ↓ denote lower metrics indicate better performance, and arrows ↑ denote higher metrics indicate better performance. Bold values represent the best-performing results for each metric.

Method	MAE ↓	RMSE ↓	PLCC ↑	SRCC ↑
Same-input Ridge	7.286 (4.943–9.591)	9.162 (6.432–11.407)	0.745 (0.588–0.887)	0.710 (0.408–0.859)
Same-input MLP	6.732 (3.769–11.380)	10.007 (5.124–15.466)	0.691 (0.302–0.926)	0.579 (0.058–0.816)
Auxiliary direct-score head	4.580 (3.095–6.396)	6.264 (4.628–8.041)	0.890 (0.800–0.941)	0.833 (0.629–0.876)
**Full RESC**	**4.450** (3.199–6.139)	**5.870** (4.367–7.701)	**0.902** (0.837–0.944)	**0.852** (0.665–0.887)

**Table 7:** Ablation of general pose statistics and rubric-aligned geometric evidence. The three settings differ only in input evidence; model architecture and training strategy remain unchanged. Down and up arrows indicate that lower and higher values are better, respectively. The best value in each column is shown in bold.

Input Evidence	MAE ↓	RMSE ↓	PLCC ↑	SRCC ↑
General pose statistics	6.749	9.264	0.714	0.644
Rubric geometry	7.012	9.291	0.712	0.721
**General pose statistics + rubric geometry**	**4.450**	**5.870**	**0.902**	**0.852**

**Table 8:** Ablation of RESC components. Each variant removes one design from the complete framework, which is shown in the final row. Down and up arrows indicate that lower and higher values are better, respectively. The best value in each column is shown in bold.

Variant	MAE ↓	RMSE ↓	PLCC ↑	SRCC ↑
w/o error prototypes	7.546	8.614	0.696	0.645
w/o ordinal severity	5.769	6.771	0.797	0.744
w/o adaptive deduction gate	5.808	5.963	0.802	0.767
w/o monotonicity	4.503	6.076	0.880	0.804
w/o consistency	4.507	5.899	**0.908**	0.831
**RESC**	**4.450**	**5.870**	0.902	**0.852**

**Table 9:** Counterfactual directional-consistency stress test under adjacent prototype-evidence interventions.

Rubric Item	Adjacent Interventions	Item-Deduction Consistency (%)	Final-Score Consistency (%)	Maximum Reverse Score Change (Points)
Trunk lean	4763	100.00	100.00	0.0000
Knee posture	4699	100.00	100.00	0.0000
Left-elbow raise	4841	100.00	100.00	0.0000
Stroke amplitude	5169	100.00	100.00	0.0000
Hand posture	4882	100.00	100.00	0.0000
Narrow stance	4955	100.00	95.40	0.0016
Overall	29,309	100.00	99.22	0.0016

**Table 10:** Ordinal severity performance of RESC on pooled outer-LOSO test predictions.

Metric	Value
Discrete-level MAE	0.496
Exact ordinal accuracy (%)	72.76
Within-one-level accuracy (%)	85.82
Quadratic-weighted kappa (QWK)	0.631

## Data Availability

The video data and associated annotations are not publicly available because they contain recordings of human participants and are part of an ongoing study. Requests for non-commercial academic access may be directed to the first author at mfkeyman@163.com and will be considered subject to participant consent, privacy protection, and applicable institutional requirements.

## Abbreviations

The following abbreviations are used in this manuscript:

AQA	Action quality assessment
AP	Average precision
KRR	Kernel ridge regression
LOSO	Leave-one-subject-out
QWK	Quadratic-weighted Cohen’s kappa

## Appendix A. RESC Configuration

**Table A1:** Model configuration and training settings of RESC. Fold-fitted quantities were estimated exclusively from the outer-training participants.

Setting	Value or Selection Rule
Raw feature dimension	438.
PCA output and retention rule	12 dimensions, predefined for all folds; fitted within each fold using training participants only.
Encoder input dimension	22, comprising 12 PCA components and 10 directly retained rubric-specific descriptors.
Encoder dimensions	22–128–128.
Adaptive-gate dimensions	134–64–6, using the concatenated 128-dimensional latent representation and six prototype similarities; sigmoid output.
Activation	GELU in the encoder and adaptive gate.
Normalization	LayerNorm after each encoder linear layer.
Dropout	0.18, applied to the encoder.
Optimizer	AdamW.
Initial learning rate	0.001.
Weight decay	0.0002.
Batch size	16.
Maximum training epochs	240.
Early stopping	Patience 28, based on the inner-validation objective.
Early-stopping objective	Weighted inner-validation loss: deduction score 1.6; error classification 0.75; ordinal severity 0.65.
Random seed	42.
Score-loss coefficients	Deduction-chain score 2.3; auxiliary direct score 0.2.
Diagnostic-loss coefficients	Quality level 0.35; error classification 1.15.
Severity-loss coefficients	Ordinal severity 0.9; expected-severity absolute error 0.2.
Structural-loss coefficients	Score consistency 0.55; deduction-weight regularization 0.05.

**Table A2:** Compact specification and dimensional decomposition of the 438-dimensional pose feature vector.

Feature Block	Scalar Descriptors and Aggregation	Dim.
General pose	Detection confidence and body scale (4); trunk angle (1); bilateral knee angle/flexion (4); bilateral elbow angle/relative height (4); wrist, shoulder, hip, and ankle midpoint coordinates (8); normalized inter-joint distances (7). Seven temporal statistics per scalar plus four sequence summaries.	200
Trunk lean	Five shoulder–hip–knee–ankle alignment and line-deviation scalars, each summarized by nine temporal statistics.	45
Knee/support	Six lower-limb angle, flexion, and support-line scalars, each summarized by nine temporal statistics.	54
Left-elbow raise	Three relative-height and joint-angle scalars, each summarized by nine temporal statistics.	27
Stroke amplitude	Six wrist-position scalars with nine temporal statistics, plus 12 path-length, range, and displacement summaries.	66
Hand posture	Four normalized wrist–elbow distance scalars, each summarized by nine temporal statistics.	36
Narrow stance	One normalized stance-distance scalar summarized by ten temporal and validity statistics.	10
Total		438

**Table A3:** Paired participant-cluster bootstrap differences between RESC and the strongest feature-learning and structured baselines. Each interval was obtained from 10,000 resamples of the 25 outer-test participants. Positive differences favor RESC.

Baseline	ΔMAE	ΔRMSE	ΔPLCC	ΔSRCC
USDL	2.224 (−0.774–5.344)	3.525 (−0.760–7.400)	0.164 (−0.015–0.429)	0.182 (−0.062–0.402)
NS-AQA-adapted	2.259 (0.428–3.863)	2.189 (0.389–3.926)	0.100 (0.019–0.198)	0.182 (0.008–0.398)

ΔMAE and ΔRMSE are computed as baseline minus RESC; ΔPLCC and ΔSRCC are computed as RESC minus baseline. Values in parentheses are participant-cluster bootstrap 95% confidence intervals.
